# Novel Redox-Dependent Esterase Activity (EC 3.1.1.2) for DJ-1: Implications for Parkinson’s Disease

**DOI:** 10.3390/ijms17081346

**Published:** 2016-08-22

**Authors:** Emmanuel Vázquez-Mayorga, Ángel G. Díaz-Sánchez, Ruben K. Dagda, Carlos A. Domínguez-Solís, Raul Y. Dagda, Cynthia K. Coronado-Ramírez, Alejandro Martínez-Martínez

**Affiliations:** 1 Instituto de Ciencias Biomédicas, Universidad Autónoma de Ciudad Juárez, Anillo envolvente Pronaf y Estocolmo s/n, Ciudad Juarez, Chihuahua 32310, Mexico; emmanuel.vazquez@uacj.mx (E.V.-M.); al135792@alumnos.uacj.mx (C.A.D.-S.); al113722@alumnos.uacj.mx (C.K.C.-R.); 2 Department of Pharmacology, University of Nevada, Reno School of Medicine, Mailstop 318, Manville Building 19A(Office)/18(Lab), Reno, NV 89557, USA; rdagda@medicine.nevada.edu (R.K.D.); rauld@medicine.nevada.edu (R.Y.D.); 3 El Colegio de Chihuahua, Calle Partido Díaz 4723 esquina con Anillo Envolvente del Pronaf, colonia Progresista, Ciudad Juárez, Chihuahua 32310, Mexico

**Keywords:** 4-nitrophenyl acetate, human carboxyl esterase, redox sensor, oxidative stress, DJ1/PARK7, ROS, Parkinson’s disease

## Abstract

Mutations the in human *DJ-1* (*hDJ-1*) gene are associated with early-onset autosomal recessive forms of Parkinson’s disease (PD). hDJ-1/parkinsonism associated deglycase (PARK7) is a cytoprotective multi-functional protein that contains a conserved cysteine-protease domain. Given that cysteine-proteases can act on both amide and ester substrates, we surmised that hDJ-1 possessed cysteine-mediated esterase activity. To test this hypothesis, hDJ-1 was overexpressed, purified and tested for activity towards 4-nitrophenyl acetate (pNPA) as µmol of pNPA hydrolyzed/min/mg·protein (U/mg protein). hDJ-1 showed maximum reaction velocity esterase activity (*V*_max_ = 235.10 ± 12.00 U/mg protein), with a sigmoidal fit (*S*_0.5_ = 0.55 ± 0.040 mM) and apparent positive cooperativity (Hill coefficient of 2.05 ± 0.28). A PD-associated mutant of DJ-1 (M26I) lacked activity. Unlike its protease activity which is inactivated by reactive oxygen species (ROS), esterase activity of hDJ-1 is enhanced upon exposure to low concentrations of hydrogen peroxide (<10 µM) and plateaus at elevated concentrations (>100 µM) suggesting that its activity is resistant to oxidative stress. Esterase activity of DJ-1 requires oxidation of catalytic cysteines, as chemically protecting cysteines blocked its activity whereas an oxido-mimetic mutant of DJ-1 (C106D) exhibited robust esterase activity. Molecular docking studies suggest that C106 and L126 within its catalytic site interact with esterase substrates. Overall, our data show that hDJ-1 contains intrinsic redox-sensitive esterase activity that is abolished in a PD-associated mutant form of the hDJ-1 protein.

## 1. Introduction

Mutations in *parkinsonism associated deglycase* (*PARK7*) gene, which encodes for the human DJ-1 (hDJ-1)/PARK7 protein, are associated with autosomal recessive early-onset forms of Parkinson’s disease (PD) [1]. hDJ-1 has been described as a cytoprotective multifunctional enzyme with antioxidant, protease, glyoxalase and deglycase activities [2]. In addition, hDJ-1 has been shown to act as a transcriptional regulator that enables the activation of antioxidant responses to confer cytoprotection against oxidative stress (reviewed elsewhere [3,4]). In further support of the functional role of hDJ-1 as a protease, hDJ-1 has high structural homology to the DJ-1/ThiJ/PfpI superfamily, an evolutionarily conserved superfamily of cysteine proteases [5,6]. Interestingly, the oxidative-sensing and protease activities of DJ-1 have been suggested to require the redox-sensing catalytic cysteine residue (C106) and other hydrophilic amino acid residues (H126, E18) [5,7]. Although the requirement for a catalytic triad for several catalytic functions of DJ-1 remains highly controversial, it is clear that C106 plays critical roles in mediating multiple functions of DJ-1 [8].

The high resolution crystal structure of hDJ-1 has been solved by Wilson and colleagues [9]. hDJ-1 consists of an α/β-fold comprised of 10 α-helices and 12 β-strands with high homology to bacterial proteases PfpI. Several studies have shown that hDJ-1 possesses in vitro redox-sensitive proteolytic activity towards different peptides [8]. In addition, the proteolytic function requires the presence of C106 and dimerization of DJ-1. Moreover, hDJ-1 is a zymogen that undergoes catalytic activation via the cleavage of a small C-terminal molecular region which further increases its proteolytic activity [8]. Given that the autocatalytic cleavage of hDJ-1 is unlikely to occur [8], the endogenous protease that cleaves the zymogen of hDJ-1 remains to be identified. In addition, although the acute exposure of cells transiently expressing heterologous hDJ-1 to oxidative stress enhances the proteolytic cleavage of the zymogen of DJ-1, its proteolytic activity decreases in vitro while the cytoprotective activities of DJ-1 are increased [8], suggesting that prosurvival effects and proteolytic activity of DJ-1 are inversely related. In addition, the proteolytic substrates of DJ-1 in vivo have not been identified to date. The C106 of the catalytic triad C106/H126/E18 has been proposed to serve as a redox sensor that modulates the cytoprotective activities of DJ-1 [9]. The thiol (SH) group in C106 of hDJ-1 is a solvent-exposed chemical group that can be sequentially oxidized to sulfenic (SOH), sulfinic (SO_2_H), sulfinate (SO_2_¯) and sulfonate (SO_3_¯), respectively [9,10,11]. The oxidations of SH groups to SO_3_¯ are irreversible (e.g., DJ-1) and elicit the ubiquitin-proteasome dependent degradation of DJ-1 due to the inactivation of its cytoprotective activities caused by oxidative damage [7,12]. Given that the activated sulfur in C106 can adapt multiple conformational arrangements within the catalytic pocket, it is conceivable that the catalytic cysteine can recognize multiple substrates upon exposure to reactive oxygen species (ROS) [7,9]. It has been observed that the crystal structure of hDJ-1 contains a papain-like domain that is typical of cysteine proteases [7]. However, the fact that the spatial arrangement of the amino acids of the catalytic triad does not favor protease activity [7], it is conceivable that hDJ-1 favors catalysis of multiple substrates including esters in a similar manner to papain. In addition, as postulated elsewhere [7], it is not known whether the catalytic site of DJ-1 is capable of performing other enzymatic activities besides its known protease, deglycase and glyoxalase activities.

A variety of cysteine proteases, like papain, are known to possess other enzymatic activities including esterase activity [13,14,15]. It is worth noting that the superfamily of DJ-1/ThiJ/PfpI cysteine proteases contain a papain-like domain with the potential for hydrolyzing amide (peptide) or ester bonds. Therefore, based on this observation, we surmised that members of the superfamily of DJ-1/ThiJ/PfpI, including hDJ-1, contain intrinsic esterase activity. Furthermore, the temporal dynamics of the oxidation of the catalytic cysteine in relation to the catalytic activity of hDJ-1 has yet to be explored [16]. In this study, we show for the first time that hDJ-1 possesses intrinsic esterase activity with positive cooperativity. The esterase activity of hDJ-1 is robustly enhanced upon exposure to low micromolar concentrations (i.e., <10 µM) of hydrogen peroxide (H_2_O_2_) and it is extremely resistant to inactivation by high levels of the oxidant. The preincubation of hDJ-1 with the SH-protecting chemical iodoacetamide (IAA) completely abolishes the redox-sensitive esterase activity suggesting that the oxidation of solvent-exposed cysteines is required for hDJ-1 esterase activity. Mechanistically, the esterase activity of hDJ-1 requires oxidation as an oxido-mimetic mutant of hDJ-1 (C016D) is constitutively active towards 4-nitrophenyl acetate (pNPA) whereas a PD-associated mutant, known to reduce oxidation of cysteines in hDJ-1, suppresses its esterase activity. Finally, molecular docking studies suggest that C106 and L126 within the catalytic site interact with esterase substrates. In aggregate, our data suggest that the esterase activity of DJ-1 plays a role in the cytoprotective role of DJ-1 and, conceivable in the etiology of PD.

## 2. Results

### 2.1. Cloning and Overexpression of Recombinant hDJ-1 and Its Mutants C106D and M26I

**Figure 1:**
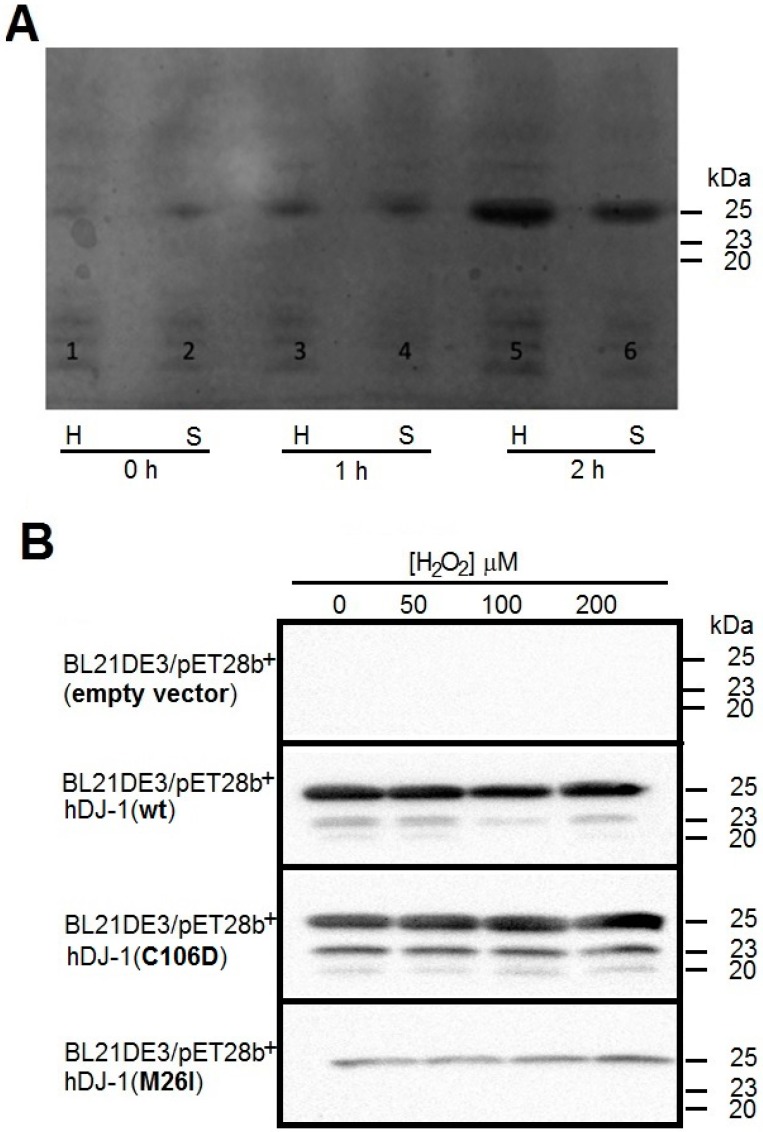
Purification and proteolytic processing of exogenous hDJ-1 from *Escherichia coli*. (**A**) Coomassie staining of protein in crude homogenates and soluble fractions derived from *E. coli* transformed with human DJ-1 (BL21DE3/hDJ-1) and incubated with 100 µM isopropyl β-d-1-thiogalactopyranoside (IPTG) for 0, 1, and 2 h. Cell lysates were loaded (100 μg protein) and separated in 15% acrylamide/bis-acrylamide by sodium dodecyl sulfate-polyacrylamide gel electrophoresis (SDS-PAGE). H: crude homogenate, S: soluble fraction derived from the crude homogenate; (**B**) Western blot of lysates from untransformed (BL21DE3) or transformed bacteria with either a wild-type (wt, BL21DE3/hDJ-1), a PD associated mutant of DJ-1 (BL21DE3/hDJ-1 M26I) or a mutant of DJ-1 that mimic oxidized cysteine-SO_2_H (BL21DE3/hDJ-1 C106D) at the indicated concentration of hydrogen peroxide (H_2_O_2_). Supernatants from lysed bacteria were purified by using a Ni^2+^-Sepharose HiTrap™ high performance (HP) column, subjected to SDS-PAGE, and immunoblotted with rabbit polyclonal antiDJ-1 antibody 1:10,000 (anti-PARK7/DJ-1 antibody [EP2815Y]). Molecular weights are indicated on the right of the image in kDa.

To test for esterase activity of hDJ-1 in vitro, human DJ-1 His-tagged, as well as mutants C106D and M26I were expressed in *Escherichia coli* BL21 (DE3) and purified in accordance to well-established protocols [8]. The temporal dynamics of the expression of wild-type (wt) hDJ-1, mutant C106D and M26I in *E. coli* BL21 (DE3) were monitored by Coomassie staining analyses of crude homogenates collected at 0, 1, and 2 h following induction with 100 µM of isopropyl β-d-1-thiogalactopyranoside (IPTG). Coomassie staining analyses demonstrated that recombinant hDJ-1 is expressed in *E. coli* with a molecular weight of 25 kDa (N-terminally His tagged + full-length hDJ-1) in the soluble fraction (Figure 1A). The supernatants of crude homogenates from all aforementioned clones were subjected to downstream purification by using a Ni^2+^-affinity column which enriched hDJ-1 and its mutant forms (C106D and M26I) to high purity as determined by Western blotting by employing a rabbit polyclonal anti-DJ-1 antibody raised against a synthetic peptide corresponding to residues near the N-terminus of DJ-1 (anti-hDJ-1/PARK7 antibody [EP2815Y]). Western blot analyses of purified eluted fractions (Figure 1B and Figure S1) revealed three immunoreactive bands (25, 23, and 20 kDa) consistent for full-length and proteolytically processed forms of hDJ-1(wt) and hDJ-1(C106D), whereas a single immunoreactive band (25 kDa) was observed for hDJ1(M26I). Unexpectedly, treating hDJ-1-expressing *E. coli* with H_2_O_2_ did not further increase the modest proteolytic cleavage of hDJ-1 in IPTG-induced *E. coli* (Figure 1B). In summary, our Western blot analysis confirmed successful inducible overexpression, solubility, and enrichment of hDJ-1(wt), hDJ-1(C106D) and hDJ-1(M26I) from transformed *E. coli*.

### 2.2. Kinetics Parameters of Esterase Activity of hDJ-1 and Its Enhancement by Oxidation

**Figure 2:**
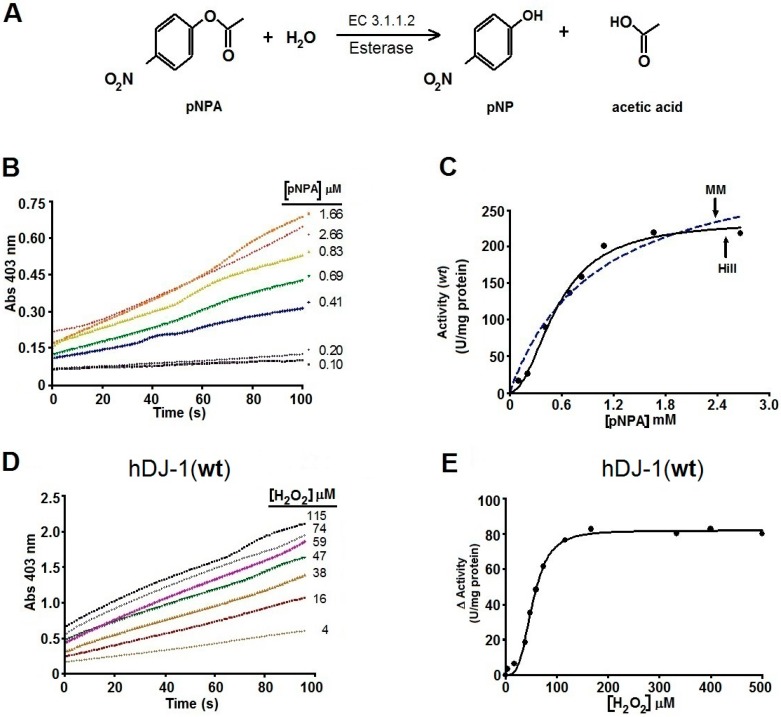
hDJ-1 contains intrinsic esterase activity that is enhanced by exposure to reactive oxygen species (ROS). (**A**) Schematic showing the hydrolysis of 4-nitrophenyl acetate (pNPA) by esterase to 4***-***nitrophenol (pNP) and acetate (EC 3.1.1.2); (**B**) Representative enzymatic kinetic time course for hDJ-1 esterase activity as spectrophotometrically monitored by the appearance of pNP at optical density (OD) of 403 nm at the indicated substrate concentrations (µM). To obtain specific esterase activity associated with hDJ-1, the absorbance values from control reactions that lacked hDJ-1 but contained H_2_O_2_, were subtracted from the values derived from the reactions containing hDJ-1 and H_2_O_2_. The enzymatic kinetic assay shown is representative of three experiments with similar results; (**C**) Representative enzymatic kinetic activity curve of hDJ-1(wt) (U/mg protein) based on the initial velocities (*V*_0_) obtained from the data shown in B, demonstrates a better sigmoidal fit for hDJ-1(wt) based on the Hill kinetic model (solid line, Equation (1) in methods) than a Michaelis–Menten fit (dashed line, Equation (2) in methods). *V*_max_ = 235.10 ± 12.00 µmol of pNPA hydrolyzed/min/mg protein; *S*_0.5_ = 0.55 ± 0.040 mM; Hill coefficient *(h*) = 2.05 ± 0.28; (**D**) Representative enzyme kinetic trends of the esterase activity of recombinant hDJ-1, pretreated with the indicated increasing concentrations of H_2_O_2_, were obtained by spectrophotometrically monitoring the appearance of pNP at 403 nm at the indicated H_2_O_2_ concentrations; (**E**) Representative enzymatic kinetic of the change (∆) activity of hDJ-1(wt) (U/mg protein) based on the initial velocities (ΔActivity = [*V*_0 with peroxide_ − *V*_0 without peroxide_]) obtained from the data shown in (**D**), shows that pretreating hDJ-1 with increasing concentrations of H_2_O_2_ (>10 µm) enhances its esterase activity and plateaus at a concentration of 100 µM.

**Figure 3:**
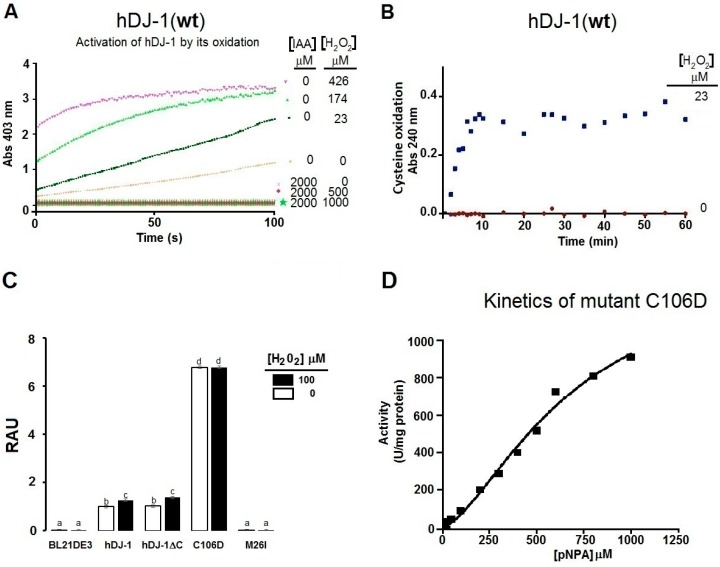
Esterase of hDJ-1 requires the oxidation of C106. (**A**) Representative enzyme kinetic trends of the esterase activity of hDJ-1, in the presence or absence of the thiol (SH)-protecting chemical iodoacetamide (IAA), co-treated with increasing concentrations of H_2_O_2_; (**B**) Oxidation of the SH group by H_2_O_2_ as induced by exposing hDJ-1 to H_2_O_2_ led to the appearance of sulfonate that was monitored at OD 240 nm; (**C**) Esterase activity expressed as relative activity units (RAU). Data are means ± standard error in untransformed bacteria (BL21DE3), purified hDJ-1(wt), oxidant-mimetic mutant of hDJ-1 (hDJ-1∆C), hDJ-1(C106D), or the PD-associated mutant of DJ-1hDJ-1(M26I) at a saturating concentration of pNPA (2 mM). Note that hDJ-1(C106D) shows enhanced esterase activity towards pNPA compared to hDJ-1(wt), whereas hDJ-1(M26I) lacks esterase activity. Multiple comparison was done by performing a one-way analysis of variance (ANOVA, *p* < 0.05) followed by post-hoc analysis, Tukey´s test. Lowercases indicates groups with statistically significant differences at *p* < 0.05; (**D**) Representative enzymatic kinetic curve of the oxido-mimetic mutant of hDJ-1(C106D) based on the initial velocities (*V*_0_) obtained from the data shown in (**C**), the kinetic parameters were: *S*_0.5_ = 676.40 ± 0.15 μM; *h* = 1.59 ± 0.15; *V*_max_ = 1434 ± 183 U/mg protein.

By using pNPA as an in vitro substrate for esterase activity (EC 3.1.1.2), colorimetric assays showed that hDJ-1 possesses strong esterase activity as monitored by the release of 4-nitro-phenoxide (pNP) induced by the hydrolysis of the ester bond (Figure 2A). The esterase activity of hDJ-1 follows a pseudo-first order kinetics (Figure 2B). The esterase activity of hDJ-1 is consistent with a sigmoidal saturation (Figure 2C, dashed line). However, upon re-examination of the saturation enzyme kinetic assays, the enzyme kinetic curve, based on the initial velocities (*V*_0_), followed a sigmoidal pattern (Figure 2C, solid line). Moreover, the enzyme kinetic data demonstrated that the esterase activity of hDJ-1 contains a Hill coefficient (*h*) of 2.05 ± 0.28. Interestingly, the esterase activity of hDJ-1 under different pH conditions is further enhanced beyond the physiological pH (7.4) and shows maximal esterase activity at a pH of 8.0 (Figure S2). These data suggest that an increase in ^−^OH enhances the esterase activity of hDJ-1. Post-translational processing of the zymogen of hDJ-1 to its mature form (~23 kDa) is required to enhance its proteolytic activity [8]. Contrary of the proteolytic activity, the pNPA esterase activity does not need the proteolytic cleavage of the C-terminal region of hDJ-1. This model is supported by the experiment shown in Figure 3C, where a truncated mutant of hDJ-1 (hDJ-1∆C) shows comparable esterase activity to full-length hDJ-1 (Figure 3C). Over more than 20 mutants of hDJ-1 have been linked to the onset of juvenile PD [4]. To determine the role of PD-associated mutations of hDJ-1 on its esterase activity, we measured the esterase activity in a PD-associated mutant of hDJ-1 (M26I), known to have reduced oxidation of solvent exposed cysteines but has an intact ability to dimerize [17]. In brief, we found that hDJ-1 (M26I) possessed no detectable esterase activity towards pNPA (Figure 2D). These results suggest that hDJ-1 esterase activity is compromised in PD models or PD pathogenesis.

### 2.3. The Esterase Activity of hDJ-1 Is Enhanced by Exposure to Reactive Oxygen Species 

Exposure of hDJ-1 to ROS has been shown to robustly reduce the proteolytic activity of hDJ-1 [8]. To this end, we surmised that the esterase activity of hDJ-1 is sensitive to ROS. To address this hypothesis, we assessed the esterase activity of recombinant hDJ-1 pre-incubated with increasing concentrations of H_2_O_2_ (0–500 µM). Unexpectedly, we observed that initial velocities (*V*_0_) of hDJ-1 were enhanced with increasing concentrations of H_2_O_2_ (Figure 2D,E). Moreover, the esterase activity of hDJ-1 subsequently plateaus upon exposure to very high micromolar concentrations (>100 µM) of H_2_O_2_ (Figure 2E).

### 2.4. Thiols Are Needed for the Esterase Activity of hDJ-1

The SH group of the catalytic cysteine in hDJ-1(C106) is highly sensitive to oxidation [11,18]. We surmised that the oxidation of solvent-exposed cysteines (C53 or C106) is required for mediating the esterase activity of hDJ-1. To address this hypothesis, we incubated hDJ-1 with IAA, a chemical which alkylates SH groups in solvent-exposed cysteines and protects thiols from further oxidation. Indeed, incubating hDJ-1 with IAA completely abolished the esterase activity of hDJ-1 incubated with a low concentration of H_2_O_2_ (Figure 3A) suggesting that the oxidation of solvent-exposed cysteine residues is required for enhancing the esterase activity of hDJ-1. We then measured the oxidation of solvent-exposed cysteines in hDJ-1 due to exposure to H_2_O_2_ by measuring the absorbance of thiolates at an optical density (OD) of 240 nm [11]. Indeed, exposing hDJ-1 to H_2_O_2_ led to a time-dependent increase in the accumulation of thiolates (Figure 3B). To further determine whether the oxidation of the catalytic cysteine (C106D) is required for the esterase activity of DJ-1, we tested for esterase activity in a mutant of hDJ-1 that mimics the oxidation of a cysteine to a thiolate (sulfinate). Indeed, we observed that the oxido-mimic mutant of hDJ-1(C106D), which retains its ability to be proteolytically processed (Figure 1B), showed a robust increase in esterase activity compared to wild-type DJ-1(wt) (Figure 3C,D). All together, these results suggest that (1) the catalytic cysteine (C106) is critical for the esterase activity of hDJ-1; (2) the oxidation of its catalytic cysteine is required for an induction of its esterase activity; and (3) the PD-associated mutant (M26I) lacks esterase activity.

**Figure 4:**
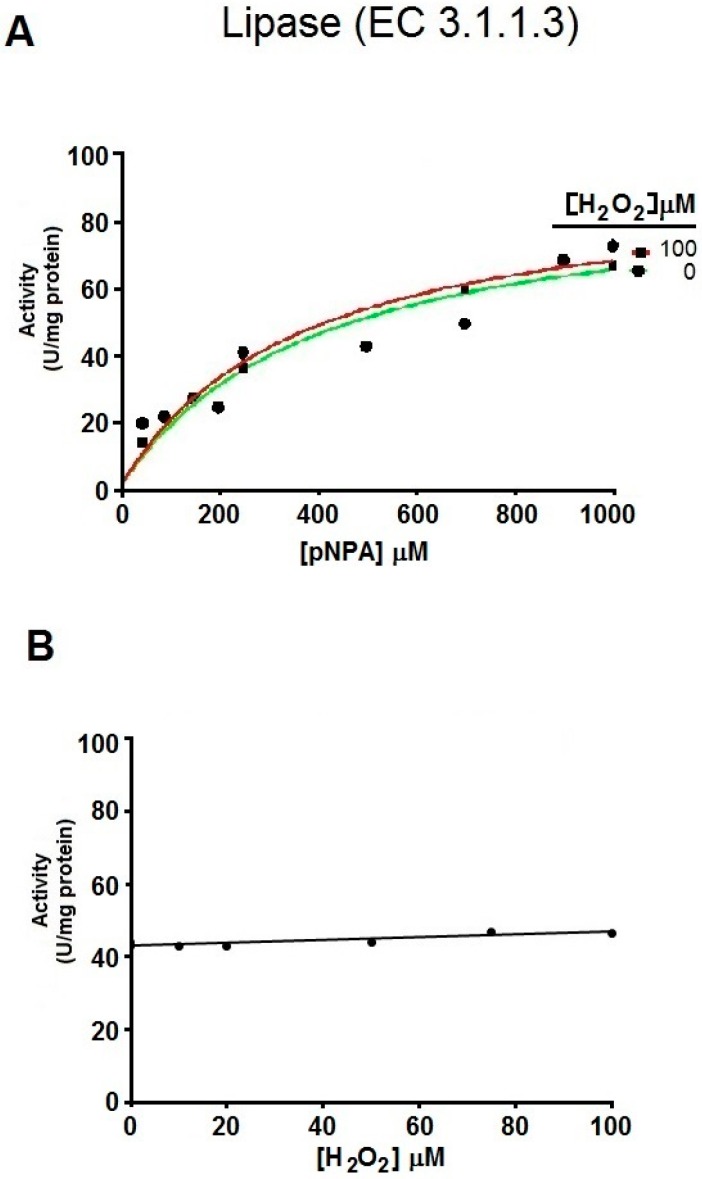

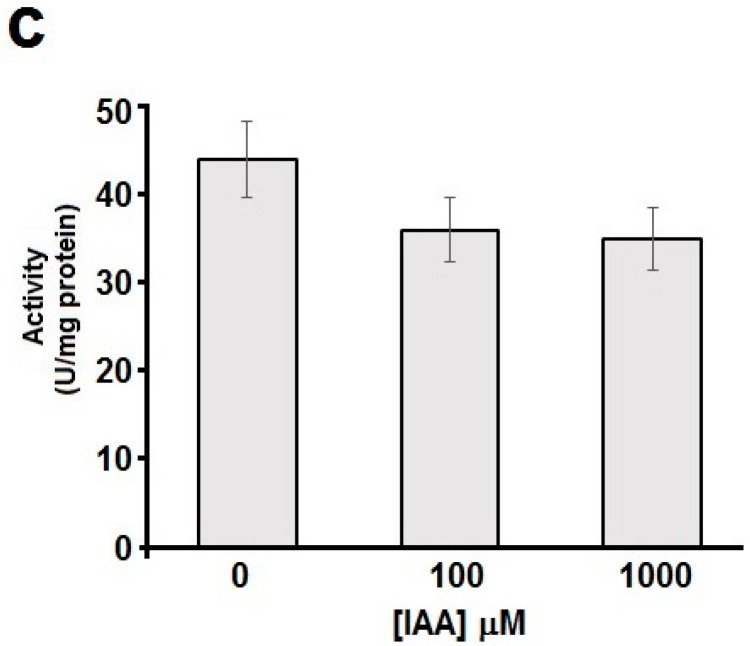
Oxidative stress does not modulate the esterase activity of pancreatic porcine lipase. (**A**) Representative enzymatic kinetic curves of esterase activity from purified porcine pancreatic lipase as spectrophotometrically monitored by the appearance of pNP (Absorbance at 403 nm) at the indicated substrate concentrations (pNPA) pretreated with vehicle buffer alone (control) or with 100 µM H_2_O_2_. Note that pre-treating pancreatic porcine lipase with H_2_O_2_ does not alter its esterase activity towards pNPA. The experiment shown in this figure is representative of two independent experiments with similar results; (**B**) Representative enzymatic kinetic curve of porcine lipase pretreated with H_2_O_2_, based on the initial velocities (*V*_0_) obtained from the data shown in A suggests that H_2_O_2_ does not modulate the esterase activity of pancreatic porcine lipase; (**C**) Representative enzymatic kinetic curves of esterase activity from purified porcine pancreatic lipase as spectrophotometrically monitored by the appearance of pNP (OD of 403 nm) at the indicated substrate concentrations (pNPA) pretreated with vehicle buffer alone (control), or with 2 mM IAA. Note that IAA treatment does not significantly decrease the esterase activity of pancreatic porcine lipase.

Next, we wanted to determine whether the redox-activated esterase activity shown by hDJ-1 is shared by other proteases that lack catalytic cysteines and which are not expected to undergo redox-sensitive activation. To address this hypothesis, we assayed the esterase activity in pancreatic lipase, a lipid esterase (EC 3.1.1.3) that lacks solvent exposed catalytic cysteines [19] and possesses the classical S-H-D catalytic triad. Indeed, treating porcine pancreatic lipase with H_2_O_2_ did not cause any induction in esterase activity (Figure 4A,B). In addition, co-treatment of pancreatic lipase with IAA did not significantly block its esterase activity (Figure 4C).

### 2.5. Molecular Docking Studies Suggest a Mechanism by Which hDJ-1 Binds to pNPA

**Figure 5:**
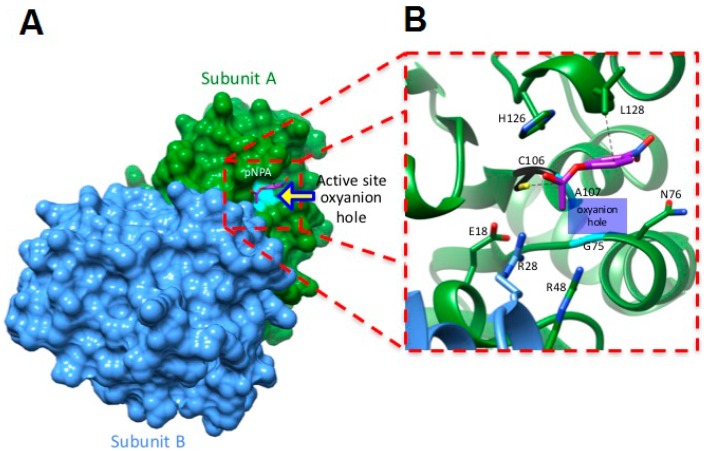
Binding of pNPA to the catalytic site of hDJ-1. (**A**) Molecular docking studies predict that pNPA binds the dimer of hDJ-1 at the oxyanion cavity of the catalytic site by forming hydrogen bonds with C106 and L128. The molecular surface of the top-ranked candidate docked structure of hDJ-1 is shown. Subunit A and B of the hDJ-1 dimer are colored green and blue, respectively; (**B**) A close up of the oxyanion site within the catalytic site of the top-ranked candidate docked structure reveals that hDJ-1 forms hydrogen bonds between pNPA via a novel catalytic dyad comprised of C106 and L128. The hatched lines denote the hydrogen bonds between atoms. For clarity, the α-helices in one of the subunits of hDJ-1 are shown in green while the α-helices belonging to the second subunit are colored blue.

Thus far, our data suggest that hDJ-1 possesses intrinsic esterase activity. To identify the interacting amino acid residues in hDJ-1 that bind to pNPA, we performed molecular docking simulations by using UCSF Chimera [20,21]. First, we employed a blind docking approach to identify any other plausible alternate binding sites in hDJ-1 that are distinct from the well-characterized catalytic cavity that harbors the C106 [8,10,11]. To this end, the “searchable” molecular space was set to encompass the complete dimer of hDJ-1. The nine best candidate docked structures, based on low annealing energies, contained pNPA docked to C106 within the catalytic site of hDJ-1. We further refined the docking of pNPA to the catalytic site of the top candidate docked structures by re-focusing the “searchable” grid to the catalytic site. In silico mapping of interacting amino acids suggests that pNPA interacts with the thiol group of C106 and with L128 within the catalytic site of the crystal structure of full-length or C-terminal truncated hDJ-1 [8] (Figure 5).

Furthermore, our molecular docking studies predict that the carbonyl group in pNPA is oriented towards the catalytic site in a manner that highly favors C106-mediated catalysis. Finally, we observed that the carbonyl oxygen of the substrate is located near the oxyanion hole comprised by peptidyl nitrogens from G75 and A107 in hDJ-1.

## 3. Discussion

### 3.1. Recombinant hDJ-1 Is Proteolytically Processed in E. coli

DJ-1 is a multifunctional redox-sensing protein with a myriad of cytoprotective functions in eukaryotic cells. Although intrinsic protease and glyoxylase activities have been reported for hDJ-1 [8,10], its endogenous proteolytic and glyoxylase substrates remain to be elucidated. In addition, it is not known whether the catalytic site of hDJ-1 is capable of performing other enzymatic activities. It is worth noting that the superfamily of DJ-1/ThiJ/PfpI cysteine proteases contain a papain-like domain with the potential for hydrolyzing amide (peptide) or ester bonds. To this end, we surmised that hDJ-1 also possesses esterase activity. To address this hypothesis, we purified recombinant hDJ-1 as well as an oxido-mimic mutant (C106D) and a PD-associated mutant (M26I), all of them derived from IPTG-induced *E. coli*. The full-length of hDJ-1 (189 aa, 20 kDa) was cloned with an N-terminal 6× histidine-tagged peptide (~1 kDa) (Figure S3). The expected molecular weight of the monomer of hDJ-1 is approximately 20 kDa, as reported elsewhere [9]. Interestingly the molecular weight of histidine-tagged hDJ-1 synthesized by *E. coli* electrophoretically migrated with an approximate molecular weight of 25 kDa (Figure 1), consistent with previous studies [22], and based on 2D gel electrophoresis data of hDJ-1 (23.2 kDa) analyzed in testis [23]. Interestingly, by employing Ni^2+^-affinity-column chromatography, western blot analyses revealed three hDJ-1 immunoreactive bands in the eluted fractions including full-length hDJ-1 (25 kDa) as well the C-terminal processed forms of the zymogen (23 kDa, 20 kDa) (Figure 1B) [8]. The fact that full and processed forms elute from the Ni^2+^-affinity-column strongly suggest that the processed region occurs in the C-terminal region, leaving intact the N-histidine tag.

### 3.2. hDJ-1 Possesses Intrinsic Esterase Activity

The sigmoidal fit of the enzyme kinetic curve associated with esterase activity of hDJ-1 (Figure 2C) is significantly different from the kinetic curves reported for its proteolytic activity [8] or glyoxalase activity [10]. Indeed, the proteolytic activity of hDJ-1 is consistent with the Michaelis–Menten model, as reported for the protease activity of hDJ-1 (*K*_m_ = 173.4 µM) towards casein [8] and for its deglycase activity (*K*_m_ = 0.44 mM) [24]. Interestingly, the esterase activity described in this paper does not fit the Michaelis–Menten model. Instead, the esterase activity fits a sigmoidal curve with positive cooperative, including the artificially-engineered mutant C106D (Figure 2E). In addition, the highly similar *S*_0.5_ (0.55 and 0.60 mM) observed for both esterase activities hDJ-1 and hDJ-1(C106D) suggest that it is unlikely due to residual enzymatic activity. This suggestion is further strengthened by the fact that a PD-associated mutant hDJ-1(M26I) [25] lacks detectable esterase activity. Finally, the enzyme kinetic data suggest that the esterase activity hDJ-1 contains a Hill coefficient of 2.05 ± 0.28 suggesting positive cooperativity and consistent for the dimeric form [9,26,27] of hDJ-1, or with the presence of two processed forms of hDJ-1 (i.e., full-length and proteolytically processed hDJ-1) [8]. Although C-terminal cleavage of DJ-1 is required for enhancing its protease activity [8], we observed that the mutant lacking the C-terminal region (hDJ-1∆C) contains comparable levels of esterase activity as hDJ-1(wt) (Figure 3C). These results suggest that the proteolytic cleavage of hDJ-1 is not essential for modulating the esterase activity of hDJ-1. Interestingly, the PD-associated mutant DJ-1(M26I), characterized to have reduced oxidation of solvent-exposed cysteines but with intact ability to dimerize, shows impaired esterase activity (Figure 3C). These results suggest that the decreased esterase activity of hDJ-1(M26I) is associated with reduced oxidation of solvent-exposed cysteines (Figure 3C) [28]. However, we recognize that additional PD-associated hDJ-1 mutants need to be screened to determine the extent by which PD-associated mutations in hDJ-1 affects its esterase activity.

### 3.3. Reactive Oxygene Species Enhance the Esterase Activity of hDJ-1

ROS-mediated activation of esterase activity is consistent with the concept that hDJ-1 is a redox-sensing cytoprotective enzyme [8]. However, unlike the esterase activity reported in this study, the protease activity of hDJ-1 is sensitive to ROS-mediated oxidation, presumably due to the oxidation of the SH group in C106, [11] which can undergo multiple oxidation states [7]. The esterase activity of hDJ-1 is elevated with increased oxidation of SH groups in solvent-exposed cysteines whereas its esterase activity is barely noticeable in the absence of oxidation (Figure 2B and Figure 3A,C). However, unlike its protease activity which is extremely labile to ROS exposure, DJ-1 esterase activity plateaus upon exposure to micromolar concentrations of H_2_O_2_ (Figure 2E). Similarly, a basic environment increases the esterase activity of hDJ-1, showing maximal esterase activity at a pH above 8.0 (Figure S2). This unexpected but intriguing data suggest that hDJ-1 possesses intrinsic esterase activity that is highly resistant to ROS and high pH (Figure 2 and Figure S2).

Given the inverse relationship in its prosurvival and protease activities when hDJ-1 is exposed to micromolar concentrations of H_2_O_2_ [8], it is likely that its protease activity does not participate in cytoprotective functions of hDJ-1 in acutely, or chronically stressed cells [29,30]. Although hDJ-1 is proteolytically cleaved in response to cellular stress, it is likely that C-terminal cleavage of hDJ-1 represents a response to compensate for the loss of protease activity caused by exposure to ROS. One limitation of this study is the inability to infer a physiological role of esterase activity of hDJ-1 based on in vitro studies. Nevertheless, this report warrants future studies to determine whether the esterase activity of hDJ-1 plays a cytoprotective role in neurons or whether its newfound esterase activity can survive the harsh oxidative environment in PD brain tissue and other neurodegenerative diseases [7,31].

### 3.4. Oxidation of Solvent-Exposed Cysteines Are Required for the Esterase Activity of hDJ-1

C106 in hDJ-1 has been extensively described as the catalytic residue and sensor of oxidative stress that can undergo sequential oxidation states [16,30,32]. The cysteine-SO_2_H of C106 is postulated to be reversible and required for certain catalytic functions of DJ-1 (e.g., transcriptional and chaperone activities) [30]. Our data suggest that the esterase activity of hDJ-1 requires the oxidation of cysteine-derived thiols as cotreating hDJ-1 with IAA completely blocked its esterase activity when exposed to low micromolar concentrations of H_2_O_2_ required to enhance its esterase activity (Figure 3A). In addition, the observation that increasing concentrations of H_2_O_2_ elevate the esterase activity of hDJ-1, suggests that multiple oxidative states of SH groups of solvent-exposed cysteines, modulate the speed of esterase catalysis. Previous studies suggest that C106-SO_2_¯ and cysteine-SO_3_¯ facilitate enzymatic catalysis of hDJ-1 by forming coordination distances of 1.5 and 3 Å with substrates [7]. Consistent with the concept that the oxidation of a catalytic cysteine is required for activating hDJ-1 esterase activity, cotreating a serine-directed lipase (from porcine pancreas, E.C. 3.1.1.3) that lacks a catalytic cysteine with IAA, or with increasing concentrations of H_2_O_2_, does not affect its esterase activity (Figure 4). In addition, the oxido-mimetic mutant hDJ-1(C106D) shows enhanced esterase activity to greater levels than DJ-1(wt) which is akin to the effects of H_2_O_2_-induced esterase activity of hDJ-1 (Figure 3C,D). Finally, our molecular docking studies predict that hDJ-1 can sterically accommodate esterase substrates (e.g., pNPA) within its catalytic site by forming hydrogen bonds with a novel catalytic dyad conformed by the catalytic cysteine (C106) and L128 with similar coordination distances. In aggregate, these results demonstrate that the oxidation of the solvent-exposed catalytic cysteine to a thiolate, presumably a sulfenic acid, is required for the esterase activity of hDJ-1.

### 3.5. Molecular Docking Studies Predict that C106 in hDJ-1 Mediates Catalysis of Esters

Our molecular docking studies predict that C106 acts as a catalytic residue for mediating the esterase activity of hDJ-1 (Figure 5). Our in silico studies are consistent with recent crystallographic and mutagenesis studies that suggest that the catalytic cysteine (C106) is required for the glyoxalase activities of *Arabidopsis thaliana*-derived DJ-1 and hDJ-1 [10]. Furthermore, our molecular docking studies suggest that the methyl group in L128 and SH moiety in C106 form a catalytic dyad to facilitate the catalysis of pNPA. Although our in silico studies did not reveal other interacting amino acid residues, we do not rule out the possibility that other amino acid residues such as E18, R28, R48, N76 and H126 can bind to pNPA under different conditions (i.e., changes in pH, oxidation state of thiols, salt concentration and temperature fluctuations in the system). Overall, our in silico analyses provide a compelling premise for identifying endogenous esterase substrates of hDJ-1 and other interacting residues that are required for its esterase activity to reduce lipid peroxidation removal under stress [33,34].

## 4. Materials and Methods

### 4.1. Cloning, Mutagenesis and Purification of Recombinant hDJ-1

hDJ-1 cDNA (UniProtKB-Q99497) was obtained from Genscript™ (Piscataway, NJ, USA). N-terminal 6× histidine tagged hDJ-1 (pET-28b^+^-HsDJ-1) was generated by subcloning hDJ-1 into the pET28b (Novagen^®^, Madison, WI, USA) vector using the NdeI-XhoI enzyme restriction sites. Mutant hDJ-1(C106D), hDJ-1(M26I), and hDJ-1(ΔC) were generated by site directed mutagenesis. hDJ-1(M26I) is an early-onset PD-associated mutant; hDJ-1(C106D) is an oxido-mimetic mutant; hDJ-1(ΔC) is a C-terminal truncated form of hDJ-1, which mimicking the mature form of the proteolytically cleaved zymogen. All mutants were generated by employing standard mutagenesis techniques by using the Quickchange II XL (Agilent Technology Inc., La Jolla, CA, USA) according to the manufacturer’s instructions with the exception that the KOD Hot Start master mix (EMD Millipore, Billerica, MA, USA) was used for reach mutagenesis reaction. The coding sequence, sense and anti-sense oligonucleotides used for performing site directed mutagenesis in full-plasmid of the pET-28b^+^-HsDJ-1 vector (EMD Millipore) are reported in Figure S3 and Table S1.

*E. coli* BL21 pLys-(DE3) expression strain (Agilent^®^, Santa Clara, CA, USA) were transformed either with pET-28b^+^-HsDJ-1 or with mutant hDJ-1(C106D) and hDJ-1(M26I) constructs by employing the CaCl_2_ technique followed by heat shock. The cloning of hDJ-1 is explained and the same procedure was done for each clone. In brief, 40 ng (5 µL) of pET-28b^+^-hDJ-1 were added into 50 µL of bacteria in Luria Broth (LB), gently mixed, and incubated on ice for 30 min. The bacteria were heat-shocked for 30 s at 42 °C, gently mixed, and returned on ice for 5 min. Thereafter, 950 mL of LB (0.5% Tryptone, 0.5% yeast extract, 1% *w*/*v* NaCl) were added and bacteria were allowed to grow at 250 rpm at 37 °C. After 2 h, bacteria were centrifuged at 1000 rpm (Eppendorf Centrifuge 5417R, rotor F-45-30-11) for 10 min, and the resulting cell pellet was suspended in 50 mL of LB and plated on selective solid LB-agar (2% agar, 0.5% tryptone, 0.5% yeast extract, 1% *w*/*v* NaCl) supplemented with 30 µg/mL kanamycin and incubated overnight at 37 °C. Thereafter, a colony was selected and used to inoculate LB medium (~10 mL) that was cultured for 12 h at 37 °C. An aliquot of 1.5 mL of bacteria was then centrifuged, resuspended in LB containing 50% glycerol and stored at −80 °C for long-term storage. To generate recombinant hDJ-1, 50 µL of bacteria were inoculated in 25 mL of LB medium overnight at 37 °C under a continuous agitation rate of 250 rpm. Fifteen mL of the resultant bacterial culture was then passaged onto 500 mL of fresh media and incubated in the same media conditions. When the culture reached an OD of 0.4–0.6 at 600 nm, the expression of hDJ-1 was induced by adding IPTG) to a final concentration of 100 µM and maintained 2 h at 37 °C. Cells were then harvested by centrifugation at 4500 rpm (Eppendorf Centrifuge 5417R, rotor F-45-30-11) for 10 min, and the cell pellet was resuspended in 30 µL of lysis buffer (50 mM 4-(2-hydroxyethyl)-1-piperazineethanesulfonic acid (HEPES), 50 mM KCl, 1% glycerol, 1 mM 2-mercaptoethanol, pH 6.8) and homogenized with an ultrasonic homogenizer bath for 30 min. To purify hDJ-1, the homogenate was centrifuged at 14,500 rpm (Eppendorf Centrifuge 5417R, rotor F-45-30-11) for 30 min at 4 °C. The resulting cell debris was discarded while the soluble fraction was loaded onto a Ni^2+^-Sepharose HiTrap™ HP column (GE Healthcare, Wauwatosa, WI, USA) that was previously equilibrated with lysis buffer. The column was then washed with 100 mL of lysis buffer containing 30 mM of ÿmidazole, and histidine-tagged hDJ-1 was eluted with 50 mL of elution buffer (50 mM HEPES, 50 mM KCl, 1% glycerol, 1 mM 2-mercaptoethanol, pH 6.8, 100 mM imidazole).

The purity of each fraction was analyzed by SDS-PAGE by electrophoresing the samples on a 15% acrylamide/bisacrylamide, at 70 V for 30 min and then at 100 V for 1.5 h. The purity of eluted recombinant hDJ-1 was corroborated by Western blot. Proteins in the SDS-PAGE gel was electrophoretically transferred onto a polyvinylidene difluoride (PVDF, Thermo Fisher^®^, Waltham, MA, USA) membrane at 150 mA for 35 min by using the Trans-Blot^®^ SD Semi-Dry Transfer Cell (BioRad, Hercules, CA, USA). The PVDF membrane was then blocked in 5% skim milk in PBS containing 1% Triton X-100 (PBST) for 1 h, washed extensively in PBST (5× for 5 min/wash) and incubated with a rabbit polyclonal anti-hDJ-1 antibody (1:10,000, anti-hDJ1/PARK7/ antibody [EP2815Y] ab76008, abcam, Cambridge, MA, USA) for 2 h at room temperature (RT). Following incubation with primary antibody, the PVDF membrane was washed 3× in PBST for 10 min/wash, incubated for 1 h with rabbit IgG anti-donkey secondary antibody conjugated to horseradish peroxidase (1:5000 in PBST), and washed 3× in PBST (10 min/wash). The immunoreactive bands were detected by incubating the PDVF membrane with SuperSignal^®^ West Pico chemiluminescent reagent (Thermo Scientific, Waltham, MA, USA) and detected using a BioRad Versadoc^®^ Imaging system.

### 4.2. Evaluation of the Hydrolysis of pNPA by hDJ-1 and Lipase 

The in vitro esterase activity of either purified full-length hDJ-1, mutants hDJ-1(C106D), hDJ-1(M26I), and hDC-1(ΔC) or pure triacylglycerol lipase (Sigma L3126) were evaluated by using the widely popular esterase substrate pNPA (Sigma N8130). The breakdown of pNPA was followed by spectrophotometry measuring the appearance of pNP at 403 nm. Briefly, 10 µL (15 µg) of hDJ-1 purified protein was added to 290 µL reaction buffer (50 mM phosphate buffer, pH 7.4), after which, 50 µL of increasing concentrations of pNPA was added to the reaction (0–1.6 mM final concentration), or maintained at a constant saturating concentration based on the experimental protocol and hypothesis tested. The absorbance at 403 nm was dynamically measured for every second for 90 s at 37 °C by using an onboard injector of FLUOstar (BMG Labtech, Allmendgruen, Ortenberg, Germany) or a Multi-mode SpectraMax M4 microplate reader (Molecular Devices, Sunnyvale, CA, USA). The spontaneous hydrolysis of pNPA in the absence of DJ-1 was used as a negative control and this background activity was subtracted from specific esterase activity associated with DJ-1. Additional negative controls conducted included enzymatic reactions containing DJ-1 but lacking pNPA. The esterase activity of purified porcine pancreatic lipase was measured as the same as for DJ-1 pretreated with vehicle buffer alone (control) or with 100 µM H_2_O_2_. Data was analyzed by fitting to Equations (1)–(3).

*V*_0_ = *V*_max_·[S]*^h^*/*S*_0.5_*^h^* + [S]*^h^*, (1)

*V*_0_ = *V*_max_·[S]/*K*_m_ + [S], (2)

∆Act = *A*_max_·[S]*^h^*/*A*_0.5_^h^ + [S]*^h^*, (3)

where *V*_0_ is the initial velocity; *V*_max_, is the maximum velocity; [S], is the substrate concentration; *h*, is the Hill coefficient; *K*_m_, is the Michaelis–Menten constant, *S*_0.5_ is the substrate concentration to accomplish half of *V*_max_; *A*_0.5_, is the hydrogen peroxide concentration that produces half of the maximum activation and ∆Act is the change in velocity.

### 4.3. Analysis of the Oxidation of hDJ-1

To analyze the role of oxidative stress on mutants and hDJ-1 esterase activity, purified hDJ-1 was exposed to increasing concentrations of H_2_O_2_ (0 to 500 µM). In brief, hDJ-1 was incubated for an hour at each concentration of H_2_O_2_ to fully ensure the oxidation of SH groups of solvent-exposed cysteines. After incubation with H_2_O_2_, the shift in UV-spectral signature associated with cysteine sulfurs was recorded [12,35]. The protonation state of thiols in hDJ-1 was monitored by measuring the absorbance at 240 nm as previously reported [35] with the following minor modifications. In brief, hDJ-1 was incubated in assay buffer (50 mM Tris, 50 mM HEPES, 50 mM MES, 50 mM KCl, 1 mM β-mercaptoethanol, pH 6.8) and with H_2_O_2_. The formation of the thiolate anion in hDJ-1 induced by exposure to H_2_O_2_ was monitored via a FLUOstar Omega™ spectrophotometer (BMG Labtech, Allmendgruen, Ortenberg, Germany) by measuring the absorbance at 240 nm and normalized with respect to 280 nm which accounts for the total thiolate concentration loaded in the incubation reaction.

### 4.4. Molecular Docking Studies

To identify molecular mechanisms by which hDJ-1 hydrolyzes pNPA, we performed molecular docking simulations by using the open source USCF Chimera algorithm [20,21] to find amino acid residues within the catalytic site of hDJ-1 that interact with pNPA. The crystal structure of hDJ-1, solved at a resolution of 1.5 Å, (PDB code: 4ZGG) was used as template and the three-dimensional structure of pNPA was downloaded from PubChem database (CID 13243). Both “receptor” (full-length or C-terminal truncated hDJ-1) and “ligand” (pNPA) structures were optimized prior to docking by employing the dockPrep function for the USCF Chimera menu [21]. In addition, all of the water molecules and the ligand were removed from hDJ-1 structure. Next, the C-terminal tail of hDJ-1 was conceptually removed as previously published [8], and the resulting molecular model was energy-minimized by using the Amber force field (Amber ff12SB) and the structure editing function of USCF-Chimera located in the tools menu. Next, a “blind” docking approach was used to identify candidate structures of hDJ-1 docked to pNPA by preparing a molecular grid that was large enough to encompass the complete structure of the dimer of hDJ-1 (size: 52.73 Å × 42.26 Å × 57.24 Å). The default docking strategy (simulated annealing) was employed for each molecular docking simulation at standard conditions. The top-ranked docked structures of hDJ-1 that contained the highest number of hits (abundance), and low binding affinities (kcal/mol) were further analyzed by using USCF Chimera.

## 5. Conclusions

The DJ-1/ThiJ/PfpI comprise an ancient superfamily of cysteine-proteases that contain a catalytic cysteine (C106 in hDJ-1) and a papain-like domain [8] with the potential to mediate proteolytic and esterase activities. Previous studies have raised the possibility that the catalytic C106 regulates additional intrinsic enzymatic activities or functional roles of hDJ-1 [7,10]. By using pNPA as an in vitro substrate for esterases, we unveiled novel intrinsic esterase activity of DJ-1 that is presumably modulated by positive cooperativity (Figure 2C). Our data is consistent with the fact that hDJ-1 is a homodimeric protein with two catalytic sites [9,11]. The value of h > 1 (2.053) indicates an apparent positive cooperativity suggesting that the activity of DJ-1 is enhanced upon binding the first substrate, thereby inducing conformational changes which increases the affinity for a second substrate. The reaction that we are describing for hDJ-1 fits to the enzyme classification (EC) number EC 3.1.1.2, which corresponds to an hydrolase (3), acting on ether bonds (.1) being carboxylic esther (.1) in phenolic esthers (EC 3.1.1.2). The systematic name, according to the IUPAC, is aryl-esther hydrolase, arylesterase, A-esterase, paraoxonase, aromatic esterase.

Although the possibility for this esterase activity was indirectly touted by one study (formation of thioesters intermediates required for its glyoxylate activity) [10], our study shows direct evidence for C106-mediated hydrolysis of esters by hDJ-1. In brief, the basal esterase activity of DJ-1 requires a homodimer conformation (Figure 2), ROS-mediated oxidation of the catalytic cysteine (C106) which robustly elevates hDJ-1’s esterase activity (Figure 2 and Figure 3). Furthermore, esterase activity may be mediated by the well-characterized catalytic pocket of hDJ-1 as suggested by our molecular docking studies (Figure 5). On the other hand, while the protease and glyoxylase activities require that a reactive SH in C106 interacts with H126, E18 and water [7,10,11], our molecular docking and biochemical analyses of the hDJ-1 mutant hDJ-1(C106D) suggests that hydrolysis of pNPA involves C106 and L128 residues and presumably requires oxidation of C106.

It is plausible both the esterase and proteolytic activities of hDJ-1 play distinct physiological roles. Like hDJ-1, other proteases are known to contain esterase and proteolytic activities that are mediated by a common catalytic site that can be allosterically modulated. Similarly, trypsin, a highly characterized pH-sensitive serine protease, possesses both intrinsic esterase and proteinase activities that can be differentially modulated by post-translational modifications [36,37]. However, our data suggest that redox-activation of esterase activity is unique to hDJ-1 (Figure 2D,E), as treating pancreatic porcine lipase, a serine protease, with H_2_O_2_ does not affect its esterase activity (Figure 4). Our findings add a major level of complexity to the physiological role of this multifunctional protein. Like many cysteine proteases, our studies suggest that hDJ-1 is a cysteine protease with more than one substrate (e.g., amide, ester) [38]. However, unlike very well-characterized cysteine proteases, it remains to be elucidated whether hydrolysis of pNPA, or mild proteolytic activity, may be a non-physiological extension or a proxy readout for distinct physiological roles of full-length hDJ-1.

It is clear that hDJ-1 is a multifunctional redox-sensing protein with broad substrate specificity to enable distinct or convergent physiological roles. On the other hand, while the proteolytic activity of full-length hDJ-1 is weak and sensitive to oxidative stress [8], our studies suggest that the esterase activity for hDJ-1 is potently activated by ROS (Figure 2D-E). However, PD mutations in DJ-1 affect its esterase activity given that a PD-associated mutant of DJ-1 was observed to lack esterase activity (Figure 3C). Hence, our study warrants future experiments to further unravel the molecular mechanisms of the esterase activity of hDJ-1 as well as the biological implications for in vivo and in the context of PD. This report lays the groundwork for future studies that examine the extent by which the esterase activity is affected in Parkinsonian models and in other PD-associated mutants of hDJ-1, in relation to its protease activity, and whether it plays a detrimental or neuroprotective role.

## Supplementary Materials

Supplementary materials can be found at www.mdpi.com/1422-0067/17/8/1346/s1.
