# Supplementary material for: Novel Redox-Dependent Esterase Activity (EC 3.1.1.2) for DJ-1: Implications for Parkinson’s Disease
Source: Int J Mol Sci. 2016 Aug 22;17(8):1346. doi: 10.3390/ijms17081346 (PMC5000742; doi:10.3390/ijms17081346)
Supplement: Supplementary file 1 [file ijms-17-01346-s001.pdf]

Supplementary Materials: Novel Redox-Dependent Esterase Activity (EC 3.1.1.2) for DJ-1: Implications for Parkinson's Disease

Emmanuel Vázquez-Mayorga, Ángel G. Díaz-Sánchez, Ruben K. Dagda, Carlos A. Domínguez-Solís, Raul Y. Dagda, Cynthia K. Coronado-Ramírez and Alejandro Martínez-Martínez

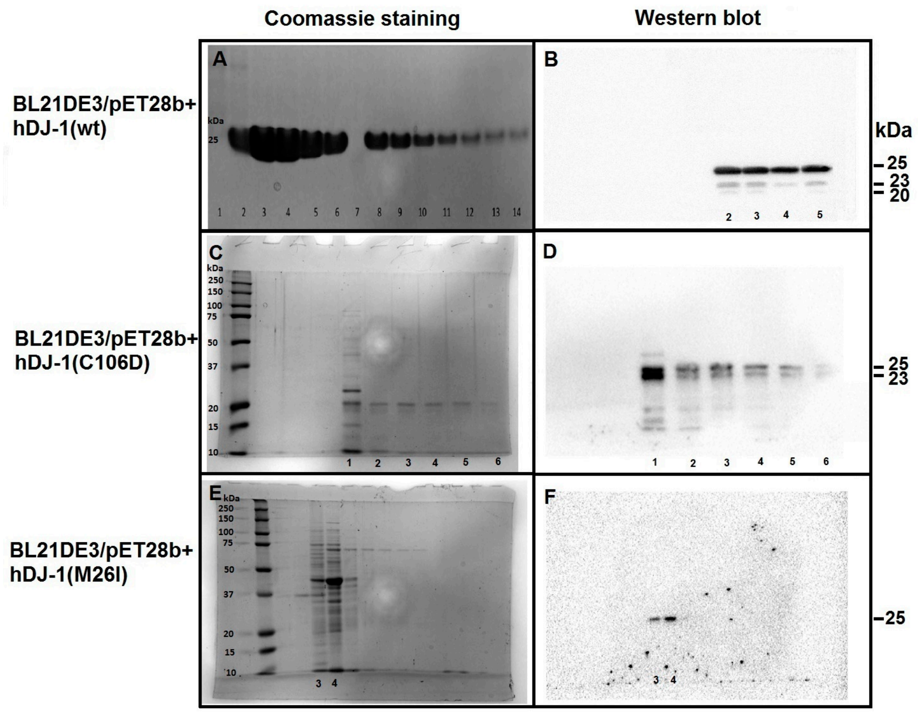

Figure S1. Sodium dodecyl sulfate-polyacrylamide gel electrophoresis (SDS-PAGE) and Western blots of purified wild-type (wt) and mutant hDJ-1 from transformed BL21(DE3) *E. coli*. (**A**–**F**) Supernatants of lysed bacteria were purified by Ni^{+2}-mediated affinity chromatography (Ni^{+2}-Sepharose HiTrap™ HP column "GE Healthcare"). Aliquots of eluted fractions (numbers below the wells) were subjected to SDS-PAGE and Commassie-stained. Molecular weight markers (MWMs) are shown on the left of each purification (**A**,**C**,**E**); (**B**,**D**,**F**) Aliquots of eluted fractions were subjected to SDS-PAGE and transferred to nitrocellulose. Anti-DJ-1 antibody was used at 1:10,000; molecular mass (kDa) is indicated on the right.

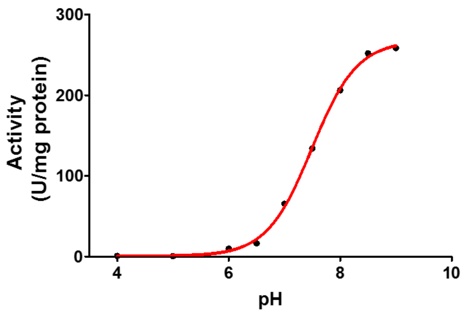

Figure S2. Human DJ-1 esterase activity is elevated at high pH levels. Representative enzymatic kinetic curves of hDJ-1 esterase activity as spectrophotometrically monitored by the appearance of 4-nitro-phenoxide (pNP) (OD of 403 nm) at the indicated pH levels. The fit curve is representative of three experiments with similar results. The data demonstrates that the esterase activity of DJ-1 increases above physiological pH (>7.4).

A) Gene Synthesis: Gene name: hDJ-1, Length: 582 bp, Vector name: pet28b⁺, Sequence:

ATGGCCTCTAAACGTGCTCTGGTTATCCTGGCGAAAGGTGCTGAAGAAATGGAAACGGT
TATCCCGGTCGATGTGATGCGTCGTGCTGGTATTAAAGTCACCGTGGCAGGTCTGGCCG
GTAAAGATCCGGTTCAGTGCTCACGTGACGTGGTTATCTGTCCGGATGCGTCGCTGGAA
GACGCCAAAAAAGAAGGCCCGTATGATGTCGTGGTTCTGCCGGGCGGTAACCTGGGTGC
GCAGAATCTGAGCGAATCTGCGGCCGTGAAAGAAATTCTGAAAGAACAAGAAAACCGCA
AAGGCCTGATTGCAGCTATCTGCGCAGGTCCGACCGCACTGCTGGCTCATGAAATCGGC
TTTGGTAGCAAAGTCACCACGCACCCGCTGGCGAAAGATAAAATGATGAACGGCGGTCA
TTATACGTACAGTGAAAATCGTGTGGAAAAAGACGGTCTGATTCTGACCAGTCGCGGCC
CGGGCACGTCCTTTGAATTCGCACTGGCTATCGTGGAAGCACTGAATGGTAAAGAAGTT
GCGGCCCAAGTCAAAGCTCCGCTGGTTCTGAAAGATTAATGACTCGAG

B) Protein product:

MASKRALVILAKGAEEMETVIPVDVMRRAGIKVTVAGLAGKDPVQCSRDVVICPDASLE
DAKKEGPYDVVVLPGGNLGAQNLSESAAVKEILKEQENRKGLIAAICAGPTALLAHEIG
FGSKVTTHPLAKDKMMNGGHYTYSENRVEKDGLILTSRGPGTSFEFALAIVEALNGKEV
AAQVKAPLVLKD

C) Blast of protein product hDJ-1

protein deglycase DJ-1 [Homo sapiens]

Sequence ID: ref|NP_009193.2| **Length:** 189 **Number of Matches:** 1

▶ See 42 more title(s)

Range 1: 1 to 189 GenPept Graphics ▼ Next Match ▲ Previous Match

Score	Expect	Method	Identities	Positives	Gaps
381 bits(978)	5e-134	Compositional matrix adjust.	189/189(100%)	189/189(100%)	0/189(0%)

```
Query  1    MASKRALVILAKGAEEMETVIPVDVMRRAGIKVTVAGLAGKDPVQCSRDVVICPDASLED  60
            MASKRALVILAKGAEEMETVIPVDVMRRAGIKVTVAGLAGKDPVQCSRDVVICPDASLED
Sbjct  1    MASKRALVILAKGAEEMETVIPVDVMRRAGIKVTVAGLAGKDPVQCSRDVVICPDASLED  60

Query  61   AKKEGPYDVVVLPGGNLGAQNLSESAAVKEILKEQENRKGLIAAICAGPTALLAHEIGFG  120
            AKKEGPYDVVVLPGGNLGAQNLSESAAVKEILKEQENRKGLIAAICAGPTALLAHEIGFG
Sbjct  61   AKKEGPYDVVVLPGGNLGAQNLSESAAVKEILKEQENRKGLIAAICAGPTALLAHEIGFG  120

Query  121  SKVTTHPLAKDKMMNGGHYTYSENRVEKDGLILTSRGPGTSFEFALAIVEALNGKEVAAQ  180
            SKVTTHPLAKDKMMNGGHYTYSENRVEKDGLILTSRGPGTSFEFALAIVEALNGKEVAAQ
Sbjct  121  SKVTTHPLAKDKMMNGGHYTYSENRVEKDGLILTSRGPGTSFEFALAIVEALNGKEVAAQ  180

Query  181  VKAPLVLKD  189
            VKAPLVLKD
Sbjct  181  VKAPLVLKD  189
```

Figure S3. Human DJ-1 coding sequence and protein product. (**A**) Coding sequence of hDJ-1 is shown as synthetized by Genscript™. N-terminal 6× histidine-tagged hDJ-1 (pET-28b⁺-HsDJ-1); (**B**) Protein product; (**C**) Blast of protein product showing the 100% identity with wild type human DJ-1.

Table S1. Sequences of primers used for full plasmid amplification site directed mutagenesis.

Mutant	Forward Primer 5′→3′	Reverse Primer 5′→3′
M26I	CCCGGTCGATGTGATACGTCGTGCTGGTA	TACCAGCACGACGTATCACATCGACCGGG
C106D	GCGGTCGGACCTGCGTCGATAGCTGCAATCAG	CTGATTGCAGCTATCGACGCAGGTCCGACCGC
ΔC	GGAAGCACTGAATGGTTAAGAAGTTGCGGC	TGGGCCGCAACTTCTTAACCATTCAGTGCTTCC

Synthesis of cDNA and cloning vector was performed by Genscript™. For the recombinant hDJ-1, the 582 bp sequence was generated and is listed in Figure S3. This sequence encodes a 189 amino acids monomer (PDB: 1j42) tagged with a 6× histidine-N-terminal tail. The DNA sequence was inserted in the pET28b⁺ vector by using the NdeI-XhoI cloning site. The mutants for the assay were synthetized by using the Recombinant *Thermococcus kodakaraensis* (KOD) DNA polymerase master mix. All mutagenesis were performed under same PCR conditions. The initial denaturation cycle was performed at 95 °C/2 min; thereafter, 30 cycles with 2 steps as follows: 95 °C/15 s; 70 °C/3.5 min; and a final extension step at 70 °C/5 min. To ensemble the reactions: 3% dimethylsulfoxyde (DMSO) was added to the reaction, 10 ng of template DNA and the corresponding primers. The primers used for the mutants were synthetized by Sigma.
